# Development and validation of a predictive nomogram for cerebral white matter hyperintensities: insights from a comprehensive clinical and laboratory analysis

**DOI:** 10.3389/fnins.2025.1642057

**Published:** 2025-11-12

**Authors:** Ning Li, Lijing Wang, Xiaoying Xu, Yadong Hu, Yajing Chen, Ye Jiang

**Affiliations:** 1 Department of Neurology, Xiongan Xuanwu Hospital, Xiongan New Area, China; 2 Department of Neurology, Affiliated Hospital of Hebei University, Baoding, China; 3 Department of Ophthalmology, Baoding No.1 Central Hospital, Baoding, China

**Keywords:** cerebral small vessel disease (CSVD), white matter hyperintensities (WMH), predictive modeling, neuroimaging, LASSO regression, nomogram, risk assessment

## Abstract

**Background:**

White matter hyperintensities (WMH) are key imaging markers of cerebral small vessel disease (CSVD), associated with cognitive decline and stroke risk. An accurate predictive model is needed for early risk assessment.

**Methods:**

This retrospective study utilized data from 587 patients undergoing cranial magnetic resonance imaging (MRI) at Hebei University’s Neurology Department. A predictive model for WMH was developed using a combination of clinical and laboratory parameters through Least Absolute Shrinkage and Selection Operator (LASSO) regression and binary logistic regression analysis. The model’s performance was evaluated using area under the receiver operating characteristic curve (AUC-ROC), calibration plots, and decision curve analysis (DCA).

**Results:**

Key predictors included age, history of stroke, hypertension, triiodothyronine levels, albumin- globulin ratio, and homocysteine. The nomogram achieved an AUC of 0.783 (95% CI: 0.738–0.829) in the training cohort and 0.762 (95% CI: 0.690–0.834) in the validation cohort. Calibration and DCA confirmed the model’s clinical applicability.

**Conclusion:**

This study presents a validated nomogram for predicting WMH, integrating clinical and biochemical markers. The model demonstrated robust predictive accuracy and potential for early risk stratification. Future studies should focus on multi-center validation and expanded risk factor inclusion.

## Introduction

Cerebral small vessel disease (CSVD), a crucial neurological disorder, presents a significant challenge to public health worldwide, especially in the aging population. CSVD is intricately linked to a range of severe outcomes, including stroke and cognitive decline, making its early detection and management imperative (Cannistraro et al., 2019; Duering et al., 2023; Wardlaw et al., 2019). White matter hyperintensities (WMH), as a key radiological hallmark of CSVD, are particularly noteworthy due to their association with increased risk of stroke, dementia, and mortality (Duering et al., 2023; Sassi, 2019; Hu et al., 2021). In T2-weighted (particularly FLAIR) magnetic resonance imaging (MRI) sequences, WMH appear as areas of high signal intensity and are typically symmetrically distributed between the cerebral hemispheres (Duering et al., 2023; Wardlaw et al., 2013). While their exact pathophysiology remains under investigation, WMH are recognized as important indicators of cerebrovascular burden and brain health.

The diagnostic capabilities of MRI are primarily confined to identifying established structural changes within the brain, indicating that early-stage WMH or subtle structural alterations might elude accurate detection. This reliance on radiological evidence alone risks the underestimation or delayed identification of WMH, especially given its frequently asymptomatic nature in the initial stages (Guan et al., 2021; Pantoni, 2010), posing challenges for timely clinical recognition in standard diagnostic procedures. This limitation highlights a clinical need for predictive models that can anticipate the presence of WMH, allowing for earlier intervention and management. Our study aims to fill this gap by constructing a predictive model for WMH, utilizing a comprehensive dataset from 587 neurology inpatients who underwent thorough cranial MRI examinations. The model incorporates a wide array of clinical and laboratory parameters, including gender, age, and histories of stroke, hypertension, diabetes, hyperlipidemia, as well as extensive laboratory findings such as complete blood count, liver and renal functions, electrolytes, blood glucose, coagulation profile, thyroid function, and homocysteine levels, totaling 88 distinct indicators. Our model offers an advanced tool for clinicians to assess and stratify WMH risk.

The development of this WMH predictive model marks a significant step forward in the early diagnosis of CSVD manifestations. It underscores the importance of integrating clinical data with radiological insights, fostering a more holistic approach in neurological care. Furthermore, the model’s capability to identify individuals at elevated risk of WMH facilitates targeted preventive strategies, potentially reducing the associated burdens of stroke and cognitive impairment.

## Materials and methods

### Study population and design

This retrospective study, conducted at a tertiary hospital in Baoding, China, aimed to identify imaging biomarkers for CSVD. The study spanned from January 2020 to June 2022. During this period, we systematically collected and analyzed existing patient records to ensure a comprehensive examination of pre-determined data. The inclusion criteria comprised patients aged 55 years or older who had undergone complete cranial MRI examinations. Patients underwent cranial MRI for routine clinical evaluations due to various neurological symptoms such as dizziness, headache, cognitive impairment, or clinical suspicion of cerebrovascular disease. The exclusion criteria were designed to omit cases that might confound the study’s outcomes, such as patients with poor-quality MRI images, a significant history of stroke defined as large territorial infarctions or hemorrhagic strokes with substantial neurological sequelae, or severe comorbid conditions. Patients with minor ischemic strokes, lacunar infarctions, or transient ischemic attacks (TIAs) who had achieved stable neurological recovery were not excluded, as the study aimed to reflect real-world inpatient populations. Among the 144 patients with a prior stroke history, 82 had lacunar infarctions, 34 had TIAs, 18 had large-vessel ischemic strokes, and 10 had remote hemorrhagic strokes. These conditions included, but were not limited to, advanced cardiac, respiratory, renal, or hepatic diseases, and tumors. Cases indicative of non-vascular origins of CSVD, like multiple sclerosis or central nervous system demyelinating diseases, were also excluded, as were those with insufficient clinical or laboratory data. To ensure the confidentiality and privacy of patient data, all records were anonymized prior to analysis, with all personal identifiers removed. The data handling process adhered strictly to data protection regulations. In line with ethical standards, the requirement for individual informed consent was waived by the Institutional Review Board due to the retrospective nature of the study. This waiver was granted as the study involved minimal risk to participants and used only anonymized data from existing records. The study protocol was thoroughly reviewed and approved by the Institutional Review Board (IRB) of Affiliated Hospital of Hebei University (Approval Number HDFYLL-KY-2023-060). The study adhered strictly to the ethical standards outlined in the Declaration of Helsinki.

### MRI acquisition and assessment

Participants underwent brain MRI examinations using a 1.5 T MRI scanner (Siemens, Munich, Germany). The standardized MRI protocol included axial T1-weighted, sagittal T2-weighted fluid-attenuated inversion recovery (FLAIR), and axial susceptibility-weighted sequences. Imaging parameters were as follows: a slice thickness of 5 mm with a 1-mm interslice gap. For T1-weighted spin echo, repetition time (TR)/echo time (TE) parameters were set at 700/11 ms. For T2-weighted fast spin echo, the TR/TE were 5200/120 ms, and for FLAIR, TR/TE and inversion time were set at 8500/127 and 2,300 ms, respectively. This imaging protocol was designed to optimally identify radiological signs of WMH. WMH, pivotal to our study, are characterized as irregularly sized signal anomalies within the white matter, distinctly hyperintense on T2-weighted MRI sequences such as FLAIR. These anomalies are non-cavitary, exhibiting a signal contrast to cerebrospinal fluid (CSF). It’s important to note that lesions located in the subcortical grey matter or brainstem are typically not classified under WMH, unless specifically mentioned. In cases where hyperintensities extend into the deep grey matter or brainstem, they are collectively referred to as subcortical hyperintensities, encompassing a broader range of abnormalities (Duering et al., 2023; Wardlaw et al., 2013). WMH were graded using the Fazekas visual scale. Both periventricular and deep WMH with a score of 1 or higher were classified into the WMH group. The identification and localization of WMH were independently assessed by two experienced neuroimaging experts, Y. H. and H. Z., who were blinded to the clinical data of the participants to minimize assessment bias. The reliability of these assessments was quantified using the intraclass correlation coefficient (ICC). A substantial agreement was indicated by an ICC value of 0.85, reflecting a high level of interrater consistency.

### Clinical blood biochemistry assessment

In our retrospective patient cohort analysis, a comprehensive dataset was meticulously compiled, including a broad spectrum of clinical parameters. This dataset incorporated 81 distinct laboratory parameters, encompassing complete blood count, renal function indicators, electrolyte levels, coagulation profiles, glucose measurements (both random and fasting), liver function tests, lipid profiles, cardiac enzyme panels, thyroid function assessments, and homocysteine levels. These extensive blood biochemistry markers provided a thorough clinical picture for each enrolled individual, covering vital health aspects.

### Clinical evaluation

A thorough clinical evaluation was conducted for each participant. This evaluation encompassed the collection of demographic information, such as age and sex, along with a detailed medical history. Emphasis was placed on key health conditions, including hypertension, diabetes, hypercholesterolemia, as well as the history of carotid artery atherosclerosis and stroke. Hypertension was defined as a systolic blood pressure ≥140 mmHg, a diastolic blood pressure ≥90 mmHg, or the use of antihypertensive medications. Diabetes was diagnosed based on fasting blood glucose levels ≥7.0 mmol/L, 2-h post-oral glucose tolerance test readings ≥11.1 mmol/L, or the use of hypoglycemic agents. Hypercholesterolemia was identified when total cholesterol or LDL cholesterol levels exceeded the upper normal range. Carotid atherosclerosis was determined by a history of the condition or evidence of increased intima-media thickness or the development of plaques in the carotid arteries, as indicated by carotid ultrasound examination.

### Statistical analysis

In this study, a cohort of 587 patients was stratified into two datasets for the development of a WMH prediction model: a training dataset of 412 patients and a validation dataset of 175 patients, following a 7:3 allocation ratio. This strategic division was pivotal for robust model training and subsequent validation. For the WMH prediction model, continuous variables were transformed into categorical forms, a method widely endorsed in risk prediction research for its interpretability and generalizability (Barrio et al., 2017; Bennette and Vickers, 2012). This approach also helped minimize the influence of outliers and address the large differences in measurement units among heterogeneous variables, thereby improving model stability and comparability across predictors. This categorization process, often necessary for clinical data, facilitates more straightforward interpretation and applicability in a clinical setting. In defining cutoffs for these variables, R software was utilized to guide the process. In instances where specific cutoffs were not predefined, variables were pragmatically classified into binary or trinary categories. Categorical variables were then presented as frequencies and percentages. To compare baseline characteristics between the WMH-positive and WMH-negative groups, appropriate statistical tests were employed based on the nature of the data. Categorical variables were analyzed using *χ*^2^ tests or Fisher’s exact tests, as appropriate. The training dataset underwent variable selection through Least Absolute Shrinkage and Selection Operator (LASSO) regression. This technique, supported by cross-validation, is instrumental in reducing overfitting by selecting a subset of relevant predictors. The optimal lambda value was determined using the lambda.1SE criterion, which is associated with the most regularized model within one standard error of the minimum cross-validation error. Following this, significant variables identified in the LASSO regression were incorporated into a binary logistic regression model to ascertain independent predictors of WMH. A nomogram was then constructed based on these predictors to visually represent the risk factors and their relative weights in predicting WMH. The efficacy of the nomogram was assessed using receiver operating characteristic curve (ROC) analysis to measure the area under the curve (AUC). Calibration plots were also generated, comparing the predicted probabilities against actual outcomes to evaluate the model’s accuracy. Decision curve analysis (DCA) was further conducted to determine the clinical utility of the model, examining the net benefits across different threshold probabilities. All statistical analyses were conducted using R software (version 4.3.0), and a *p*-value of less than 0.05 was considered statistically significant.

## Results

### Baseline characteristics

**Figure 1:**
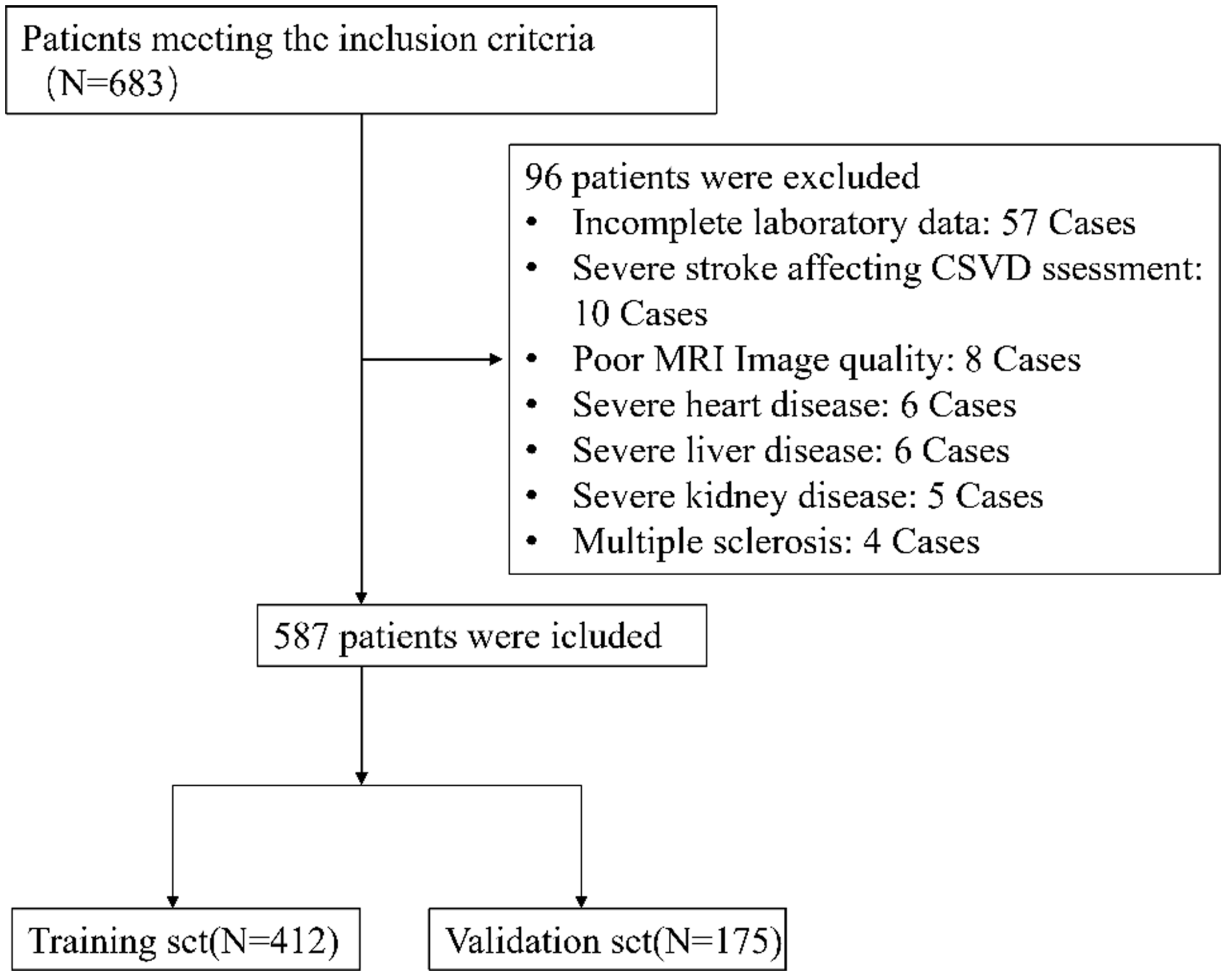
Flow diagram of the selection of eligible patients.

Between January 2020 and June 2022, a total of 683 patients were initially screened for eligibility. After applying exclusion criteria, 96 patients were excluded, resulting in 587 eligible participants (see Figure 1). These were divided into a training set (*n* = 412) and a validation set (*n* = 175). Baseline characteristics of the WMH group (*n* = 200) and non-WMH group (*n* = 387) are summarized in Table 1. Significant differences (*p* < 0.05) were found in 31 out of 88 clinical and biochemical variables. Compared with the non-WMH group, patients in the WMH group were generally older and had a higher prevalence of vascular risk factors, including hypertension, stroke history, and carotid atherosclerosis. They also showed differences in renal function, lipid profile, thyroid function, and inflammatory indices. The remaining 57 variables did not show significant intergroup differences. Detailed comparisons are presented in Table 1.

**Table 1:** Baseline characteristics: comparing wmh patients with non-WMH patients.

Variables	Total (*n* = 587)	Non-WMH (*n* = 387)	WMH (*n* = 200)	*p*
Sex, *n* (%)				0.003
Female	307 (52)	220 (57)	87 (44)	
Male	280 (48)	167 (43)	113 (56)	
Age (years), *n* (%)				< 0.001
≤68	421 (72)	306 (79)	115 (57)	
>68	166 (28)	81 (21)	85 (42)	
History of stroke, *n* (%)				< 0.001
NO	443 (75)	320 (83)	123 (62)	
YES	144 (25)	67 (17)	77 (38)	
Carotid atherosclerosis, *n* (%)				< 0.001
NO	245 (42)	181 (47)	64 (32)	
YES	342 (58)	206 (53)	136 (68)	
Hypertension stage, *n* (%)				< 0.001
Normal	189 (32)	155 (40)	34 (17)	
Level 1	109 (19)	81 (21)	28 (14)	
Level 2	251 (43)	131 (34)	120 (60)	
Level 3	38 (6)	20 (5)	18 (9)	
Hyperlipidemia, *n* (%)				0.005
NO	382 (65)	236 (61)	146 (73)	
YES	205 (35)	151 (39)	54 (27)	
Urea (mmol/L), *n* (%)				0.017
≤298	303 (52)	214 (55)	89 (44)	
>298	284 (48)	173 (45)	111 (56)	
Creatinine (μmol/L), *n* (%)				< 0.001
≤80	490 (83)	344 (89)	146 (73)	
>80	97 (17)	43 (11)	54 (27)	
Lymphocyte percentage, *n* (%)				0.048
≤24	294 (50)	182 (47)	112 (56)	
>24	293 (50)	205 (53)	88 (44)	
Monocyte count, *n* (%)				0.023
≤0.44	295 (50)	208 (54)	87 (44)	
>0.44	292 (50)	179 (46)	113 (56)	
Eosinophil count, *n* (%)				0.031
≤0.07	299 (51)	210 (54)	89 (44)	
>0.07	288 (49)	177 (46)	111 (56)	
Calcium (mmol/L), *n* (%)				0.015
≤2.32	310 (53)	190 (49)	120 (60)	
>2.32	277 (47)	197 (51)	80 (40)	
Total cholesterol (mmol/L), *n* (%)				0.037
≤4.50	295 (50)	182 (47)	113 (56)	
>4.50	292 (50)	205 (53)	87 (44)	
High-density lipoprotein (mmol/L), *n* (%)				0.013
≤1.13	306 (52)	187 (48)	119 (60)	
>1.13	281 (48)	200 (52)	81 (40)	
Low-density lipoprotein (mmol/L), *n* (%)				0.015
≤2.89	295 (50)	180 (47)	115 (57)	
>2.89	292 (50)	207 (53)	85 (42)	
Apolipoprotein A1 (g/L), *n* (%)				0.006
≤1.03	296 (50)	179 (46)	117 (58)	
>1.03	291 (50)	208 (54)	83 (42)	
Apolipoprotein B100 (g/L), *n* (%)				0.02
≤0.79	297 (51)	182 (47)	115 (57)	
>0.79	290 (49)	205 (53)	85 (42)	
Apolipoprotein E (mg/L), *n* (%)				< 0.001
≤37	296 (50)	173 (45)	123 (62)	
>37	291 (50)	214 (55)	77 (38)	
Lactate dehydrogenase (U/L), *n* (%)				0.022
≤158	307 (52)	216 (56)	91 (46)	
>158	280 (48)	171 (44)	109 (55)	
Alkaline phosphatase (U/L), *n* (%)				0.017
≤70	297 (51)	210 (54)	87 (44)	
>70	290 (49)	177 (46)	113 (56)	
Direct bilirubin (μmol/L), *n* (%)				0.004
≤3.60	302 (51)	216 (56)	86 (43)	
>3.60	285 (49)	171 (44)	114 (57)	
Albumin globulin ratio, *n* (%)				< 0.001
≤1.50	304 (52)	179 (46)	125 (62)	
>1.50	283 (48)	208 (54)	75 (38)	
Triiodothyronine (μg/dL), *n* (%)				< 0.001
≤0.64	43 (7)	13 (3)	30 (15)	
>0.64	544 (93)	374 (97)	170 (85)	
Free thyroxine (ng/mL), *n* (%)				0.005
≤1.22	304 (52)	217 (56)	87 (44)	
>1.22	283 (48)	170 (44)	113 (56)	
Homocysteine (μmol/L), *n* (%)				< 0.001
≤15	310 (53)	230 (59)	80 (40)	
>15	277 (47)	157 (41)	120 (60)	
Systemic immune-inflammatory index, *n* (%)				0.02
≤643.7	293 (50)	207 (53)	86 (43)	
>643.7	294 (50)	180 (47)	114 (57)	
Systemic inflammation response index, *n* (%)				< 0.001
≤1.23	293 (50)	213 (55)	80 (40)	
>1.23	294 (50)	174 (45)	120 (60)	
Neutrophil-to-lymphocyte ratio, *n* (%)				0.042
≤2.80	294 (50)	206 (53)	88 (44)	
>2.80	293 (50)	181 (47)	112 (56)	
Neutrophil-to-HDL ratio, *n* (%)				0.003
≤3.97	293 (50)	211 (55)	82 (41)	
>3.97	294 (50)	176 (45)	118 (59)	
Lymphocyte-to-monocyte ratio, *n* (%)				< 0.001
≤3.47	294 (50)	174 (45)	120 (60)	
>3.47	293 (50)	213 (55)	80 (40)	
Monocyte-to-HDL ratio, *n* (%)				0.003
≤0.39	293 (50)	211 (55)	82 (41)	
>0.39	294 (50)	176 (45)	118 (59)	

### Variable selection

**Figure 2:**
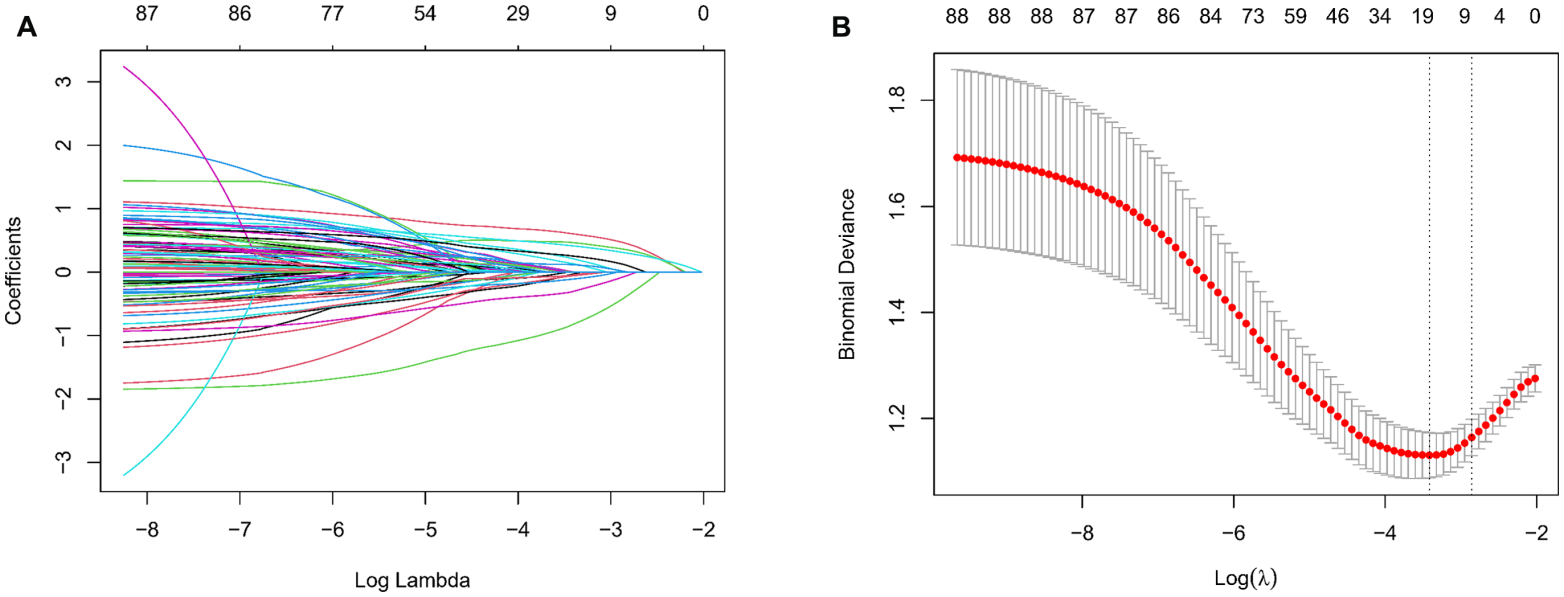
Screening of variables based on LASSO regression model in the training group (412 patients). **(A)** LASSO coefficient profiles for WMH clinical predictors. This figure presents the coefficient profiles of 88 features considered in the LASSO model for predicting the risk of WMH. The graph displays how each feature’s coefficient varies with the log of lambda (log(lambda)), demonstrating the shrinkage effect of the LASSO technique. **(B)** LASSO regression cross-validation results. This figure illustrates the evaluation of model performance under various regularization parameters *λ* through cross-validation in LASSO regression. A vertical dashed line on the left side represents λmin, which corresponds to the model with the best performance. On the right side, another vertical dashed line denotes λ1SE, representing a slightly sparser model. The numbers of selected variables are annotated above each line.

In our comprehensive analysis aimed at developing a robust diagnostic model for WMH, we meticulously compiled a dataset comprising 88 variables. This extensive dataset included crucial demographic details such as age and gender, alongside comprehensive medical histories and a wide array of laboratory tests. To distill the critical variables most predictive of WMH, we employed the LASSO (Least Absolute Shrinkage and Selection Operator) regression technique using the “glmnet package” in R. We applied a 10-fold cross-validation method to fine-tune the regularization parameter *λ*, guided by the 1SE (one standard error) criterion (Figures 2A,B). This criterion was instrumental in maintaining a balance between simplicity and predictive accuracy of the model, thereby mitigating the risk of overfitting. Through this rigorous LASSO regression approach, we identified seven pivotal indicators: age, stroke, hypertension, triiodothyronine, neutrophil-to-HDL ratio, albumin globulin ratio, and homocysteine (Table 2). Variables retaining non-zero coefficients in the LASSO model were deemed significant, suggesting a strong association with WMH. These findings offer insights into potential pathophysiological mechanisms and contribute to the advancement of diagnostic methodologies for WMH.

**Table 2:** Coefficients and lambda.1SE value of the LASSO regression.

Variable	Coefficients	Lambda.1SE
Age	0.102	0.057
History of stroke	0.082	
Hypertension	0.065	
Triiodothyronine	−0.093	
Neutrophil-to-HDL ratio	0.004	
Albumin globulin ratio	−0.014	
Homocysteine	0.024	

This table presents the LASSO regression analysis outcomes, displaying coefficients for key variables like age, stroke, hypertension, triiodothyronine, neutrophil-to-HDL ratio, albumin-globulin ratio, and homocysteine. It also includes the lambda.1SE value, crucial in model selection, indicating the model’s robustness and predictive reliability. This analysis forms a vital part of our predictive model’s development, underscoring these variables’ significance in determining the risk of WMH.

### Multivariable analyses

The binary logistic regression analysis integrated the seven variables identified via LASSO regression. This analysis revealed that six variables—age, stroke, hypertension, triiodothyronine, albumin globulin ratio, and homocysteine—were significantly associated with WMH risk (*p* < 0.05), detailed in Table 3. These findings reinforce the notion that these six variables serve as independent clinical predictors for WMH. Meanwhile, the Neutrophil-to-HDL Ratio, though included in the model, did not manifest a statistically significant correlation in this particular analysis.

**Table 3:** Binary logistic regression analysis.

	*B*	*SE*	*OR*	95% *CI*	*Z*	*P*
Age	1.008	0.261	2.74	1.64–4.57	3.864	<0.001
History of stroke	0.721	0.262	2.06	1.23–3.44	2.75	0.006
Hypertension	0.624	0.132	1.87	1.44–2.42	4.74	<0.001
Triiodothyronine	−1.582	0.441	0.21	0.09–0.49	−3.587	<0.001
Albumin globulin ratio	−0.693	0.245	0.5	0.31–0.81	−2.824	0.005
Homocysteine	0.712	0.242	2.04	1.27–3.27	2.943	0.003

This table presents the outcomes of the binary logistic regression analysis conducted to identify potential predictors of WMH. This analysis initially included seven variables identified through LASSO regression. Of these, six were retained in the final WMH diagnostic model, namely: age, history of stroke, hypertension, triiodothyronine, albumin-to-globulin ratio, and homocysteine. One variable, neutrophil-to-HDL ratio, was excluded from the final model due to its *p*-value exceeding 0.05. The table provides detailed information for each factor, including the regression coefficient (*B*), standard error (*SE*), odds ratio (*OR*), 95% confidence interval (95% CI), *Z*-value, and *p*-value. The *OR* values indicate the relative risk associated with each factor, where values above 1 suggest an increased risk for WMH, and values below 1 indicate a decreased risk.

### Predictive model development

**Figure 3:**
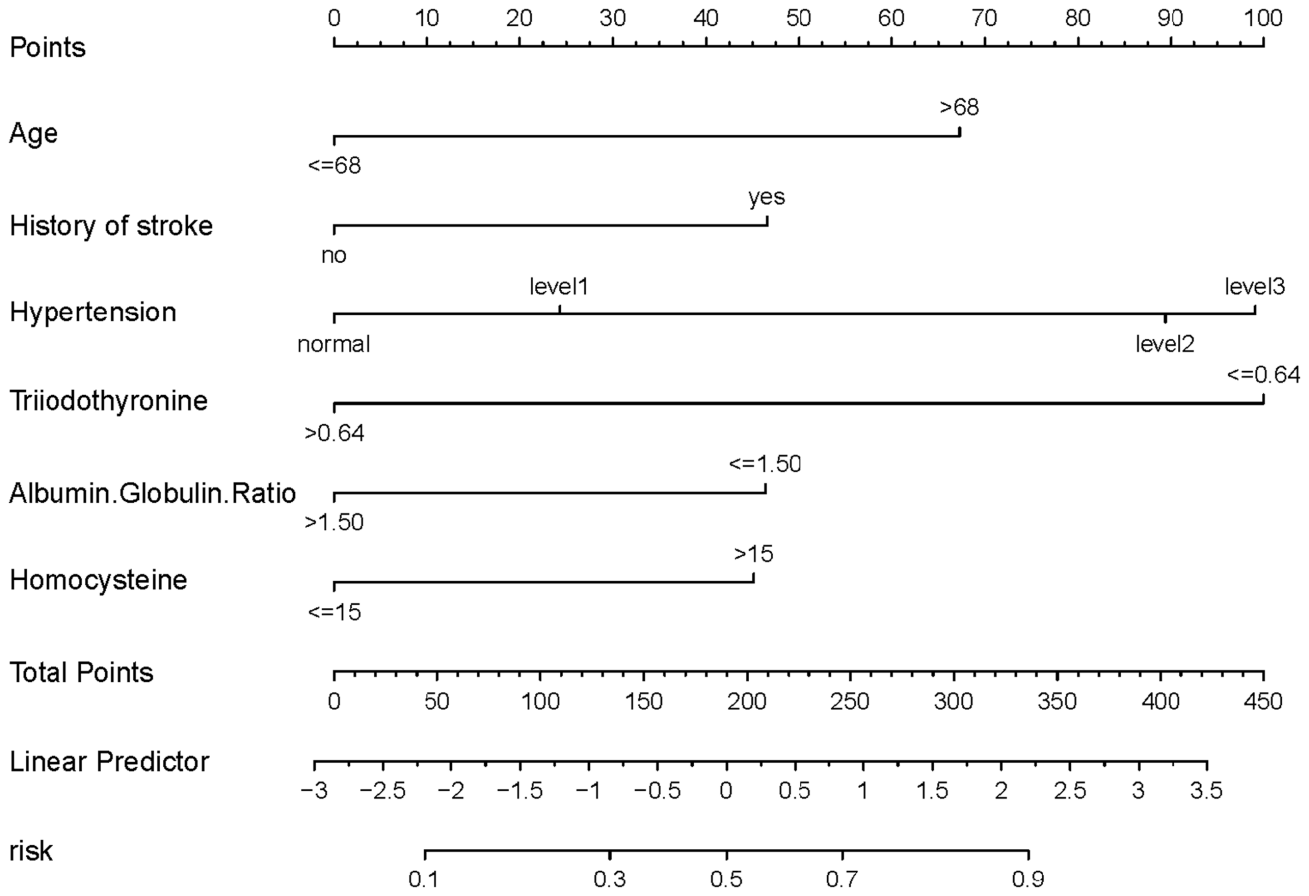
Nomogram for predicting the risk of WMH. For all patients, points are calculated based on six indicators by aligning them with corresponding point scales. The cumulative sum of points is then located on the “Total Points” axis. Subsequently, the risk of WMH, as determined by the nomogram, corresponds to the probability indicated on the “WMH” scale corresponding to the “Total Points”.

This study employed binary logistic regression analysis to identify key variables associated with the risk of WMH. These variables, namely age, stroke, hypertension, triiodothyronine, albumin globulin ratio, and homocysteine, were used to develop a predictive nomogram (Figure 3). The nomogram operates by assigning a weighted point value to each variable, reflective of their respective beta coefficients and thus their relative prognostic influence. A cumulative point total for each patient is then calculated, which correlates with an estimated probability of WMH presence. This probability is deduced from the alignment of total points with the probability scale at the nomogram’s base. Incorporating a diverse range of variables, from biochemical markers to clinical features, this nomogram facilitates a holistic assessment of WMH risk. Such a comprehensive model aids healthcare professionals in making more informed decisions about patient care and risk management strategies.

### Nomogram validation

**Figure 4:**
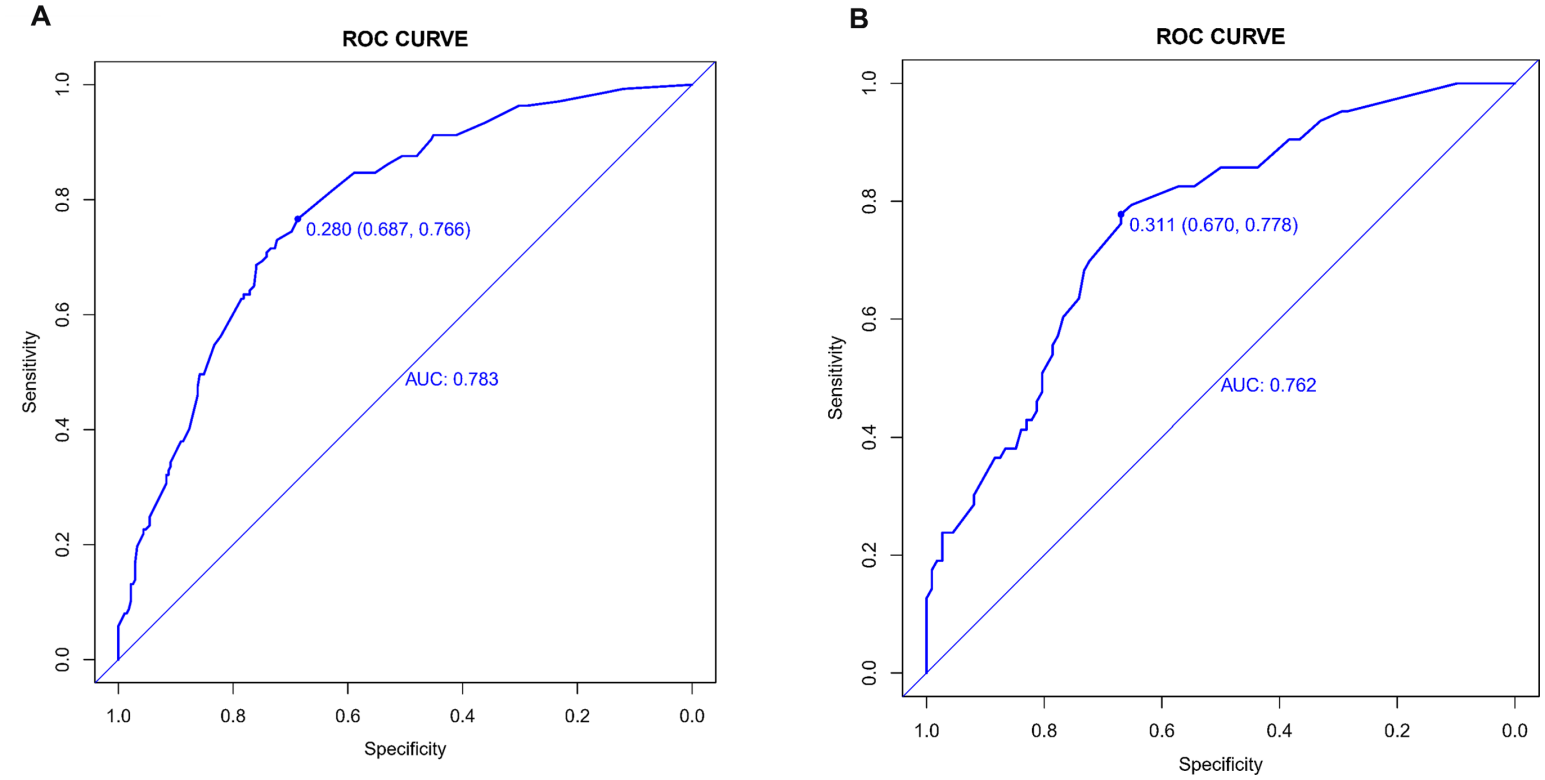
**(A,B)** Receiver operating characteristic (ROC) curves for WMH predictive model in training set **(A)** and validation set **(B)**. The ROC curves plot the sensitivity against the specificity for various threshold levels.

**Figure 5:**
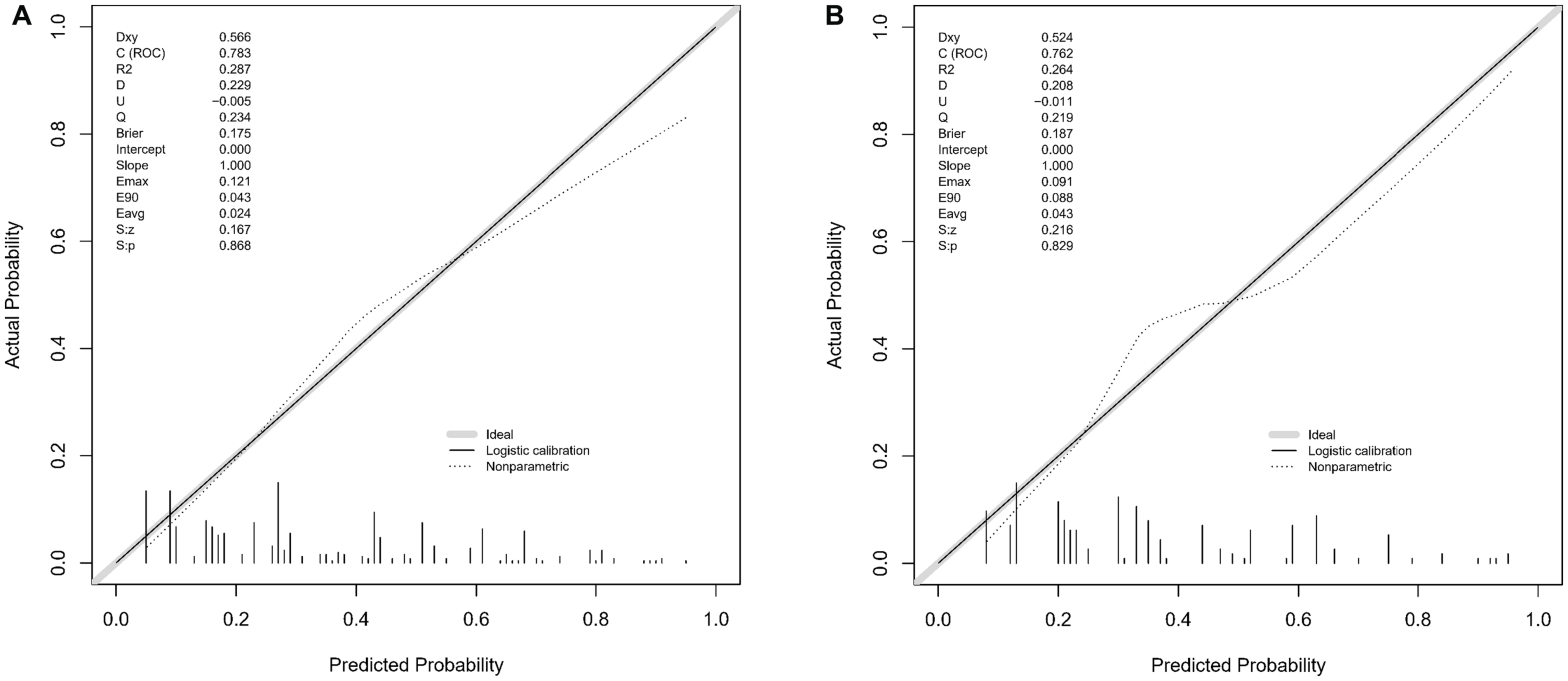
**(A,B)** These two figures present calibration plots for the WMH predictive model using the training set **(A)** and the validation set **(B)**. The calibration plots compare the predicted probabilities of WMH, as provided by the nomogram, against the actual observed frequencies.

**Figure 6:**
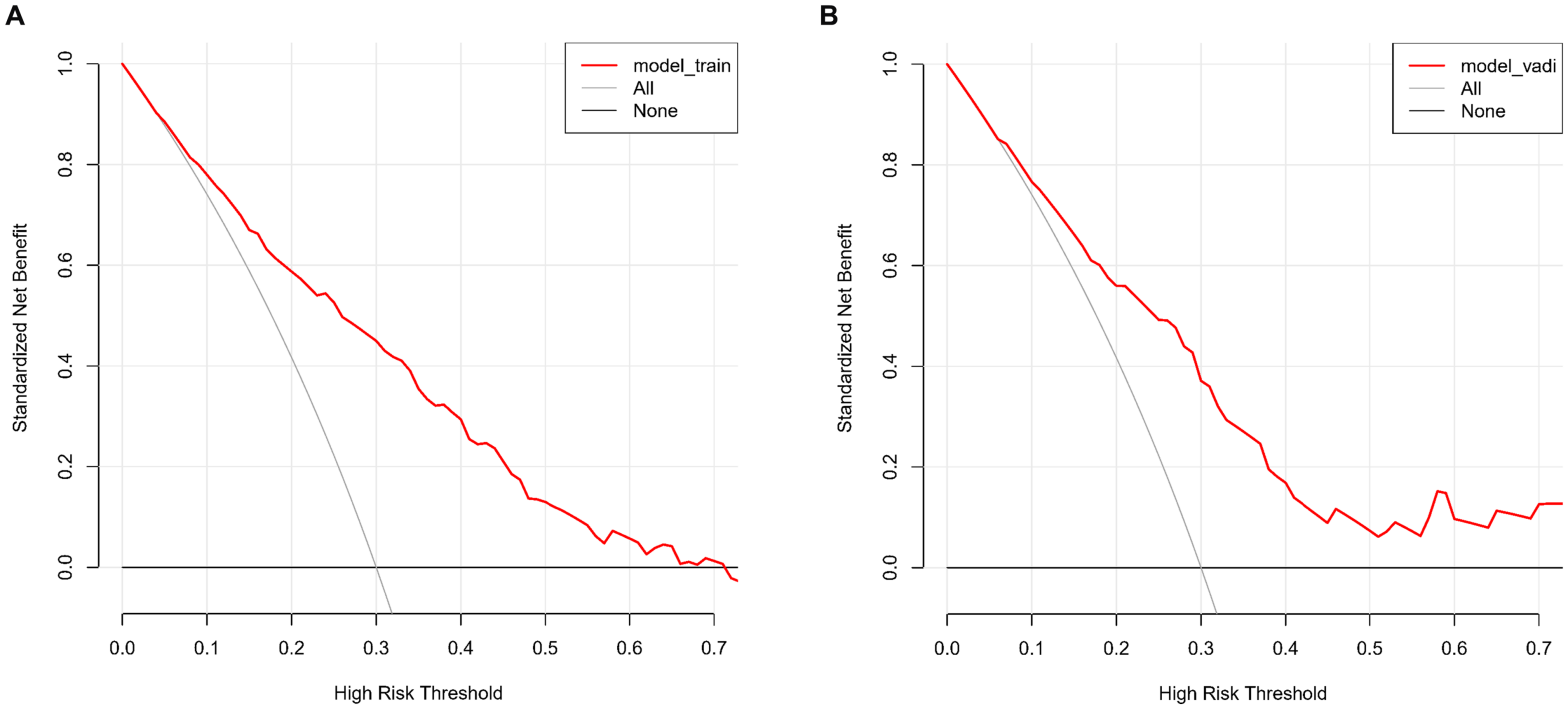
**(A,B)** These figures depict the decision curve analysis for the WMH predictive model, applied to the training set **(A)** and the validation set **(B)**. The DCA graphs display the net benefit of using the predictive model at various threshold probabilities compared to two reference strategies: treating all patients or treating none. The horizontal line in each graph represents the scenario where no patients are treated (assuming all are negative), yielding a net benefit of zero. The oblique line represents the opposite extreme, where all patients are treated (assuming all are positive). The curves of the model diverge from these extremes, indicating its clinical utility by providing a balance between the benefits and drawbacks of treatment decisions based on the model’s predictions.

The WMH risk prediction nomogram underwent rigorous validation, incorporating receiver operating characteristic (ROC) curve analysis, calibration plots, and decision curve analysis (DCA) for both training and validation cohorts. The ROC curve analysis (Figures 4A,B) demonstrated an AUC of 0.783 (95% CI: 0.738–0.829) for the training set and 0.762 (95% CI: 0.690–0.834) for the validation set, indicating the nomogram’s substantial discriminative ability for WMH risk. The sensitivity and specificity in the training set were 68.7 and 76.6%, respectively, while in the validation set, these values were 67.0 and 77.8% (Figures 4A,B). Calibration plots (Figures 5A,B) showed excellent alignment between predicted and observed outcomes, with intercepts and slopes at 0.000 and 1.000 respectively, highlighting the nomogram’s accurate predictions. The decision curve analysis (Figures 6A,B) illustrated the clinical utility of the nomogram, deviating significantly from extreme baselines and suggesting its value in improving clinical decision-making over standard care approaches. These combined validation methods underscore the nomogram’s precision and practicality in clinical settings, affirming its role as an effective tool in WMH risk prediction and management.

## Discussion

In this study, we successfully developed a predictive model for WMHs, based on a combination of clinical and laboratory parameters. Our model incorporates factors such as age, history of stroke, hypertension, triiodothyronine levels, albumin-globulin ratio, and homocysteine levels, all of which demonstrated significant associations with WMH occurrence. These findings highlight the clinical value of integrating metabolic and vascular factors for early CSVD risk assessment.

Recent nomogram studies have modeled CSVD or its subtypes using clinical and biochemical markers—including age, hypertension, creatinine or homocysteine, inflammatory indices, and previous cerebrovascular events—with reported AUCs ranging from 0.70 to 0.86 (Li et al., 2024a,b,c; Li et al., 2024). Compared with these works, our model specifically targets WMHs rather than overall CSVD burden or cognitive outcomes. Moreover, it introduces triiodothyronine (T3) as an endocrine predictor, thereby extending prior approaches by adding an endocrine dimension to CSVD risk stratification.

A novel aspect of our study is the elucidation of reduced triiodothyronine levels as a predictor of WMHs. Prior studies have established a link between thyroid dysfunction and CSVD (Zhang et al., 2017; Chu et al., 2022); however, the specific association between triiodothyronine levels and WMH risk has not been extensively explored. Our findings suggest a complex interplay between thyroid function and cerebral microvascular integrity, reinforcing the need for further investigation into the endocrine system’s role in microvascular brain injury (Guo et al., 2022). Recent studies have supported this connection, including a Mendelian-randomization analysis linking thyroid parameters to MRI markers of small-vessel disease (Tian et al., 2023) and a neuroimaging review outlining thyroid-hormone action in the human brain (Salas-Lucia, 2024). Low triiodothyronine levels may reflect a hypometabolic and hypoperfusion state that impairs myelin repair and exacerbates small-vessel injury, consistent with recent clinical evidence linking thyroid hormone levels to WMH burden (Xing et al., 2022). Mechanistically, reduced triiodothyronine (T3) may impair cerebral microvascular function by lowering endothelial nitric-oxide bioavailability, reducing cerebral perfusion, and limiting oligodendrocyte-mediated myelin repair, thereby increasing susceptibility to blood–brain barrier disruption and ischemic demyelination. The albumin to globulin ratio (AGR) emerged as a significant predictor in our study, underscoring the impact of systemic health on cerebral microvascular integrity. Previous research has demonstrated a correlation between lower AGR levels and cognitive decline in elderly populations, as well as its prognostic value in acute ischemic stroke (Maeda et al., 2019; Yang et al., 2022). Additionally, AGR has been associated with clinical outcomes in acute ischemic stroke, further reinforcing its link to systemic inflammation and nutritional status in cerebrovascular health (Wang et al., 2023). A lower AGR likely reflects systemic inflammation and reduced antioxidant capacity, which can promote endothelial dysfunction and microvascular damage. These findings collectively support the clinical relevance of AGR as a marker of systemic health and its potential utility in predicting WMH risk. Homocysteine, as a significant biomarker in our predictive model, aligns with existing research on CSVD. Previous studies have consistently demonstrated a correlation between elevated homocysteine levels and the development of CSVD, which is intrinsically linked to WMH (Staszewski et al., 2018; Nam et al., 2019). Mechanistically, homocysteine induces oxidative stress, endothelial injury, and disruption of the blood–brain barrier, all of which can accelerate white matter damage. This correlation positions homocysteine as a potential early indicator for WMH, offering valuable insights for detection and intervention strategies (Yuan et al., 2022). In addition to homocysteine, our predictive model identified hypertension, age, and a history of stroke as significant predictors, which is consistent with the current understanding of CSVD pathophysiology. These factors contribute to chronic hypoperfusion, vascular stiffening, and impaired autoregulation, further predisposing the white matter to ischemic injury. Hypertension is known to exacerbate white matter lesion burden, contributing to the development of WMH (Maniega et al., 2017; Solé-Guardia et al., 2023). Similarly, age-related changes in cerebral vessels are recognized as key factors in WMH pathogenesis (Li et al., 2020; Markus and de Erik Leeuw, 2023). A history of stroke, indicative of pre-existing cerebrovascular pathology, suggests a predisposition to further vascular injury and WMH development (Cannistraro et al., 2019; Pasi and Cordonnier, 2020). From a pathophysiological perspective, elevated homocysteine and lower AGR indicate oxidative and inflammatory stress that precipitate endothelial dysfunction. Hypertension and aging promote arteriolar remodeling and impaired autoregulation, facilitating blood–brain barrier (BBB) leakage. Reduced triiodothyronine (T3) suggests a hypometabolic state that may weaken perivascular clearance and glymphatic function while limiting remyelination. These mechanisms converge to promote diffuse white-matter injury and are jointly consistent with contemporary models of CSVD pathogenesis.

Overall, our study not only corroborates with existing literature but also offers new perspectives in understanding the relationship between WMHs and systemic health conditions. By integrating traditional and novel predictors, our model advances the understanding of WMH pathogenesis and provides a comprehensive tool for risk assessment and management.

The primary strength of our model lies in its interdisciplinary approach, integrating clinical and laboratory data for a comprehensive assessment of WMH risk. By relying on commonly assessed medical parameters, the model ensures ease of application in clinical settings, enhancing its practical utility. This integrative approach not only improves predictive accuracy but also facilitates early risk stratification, potentially aiding in timely interventions. However, this study has certain limitations. Its retrospective design may introduce selection bias, and the single-center inpatient data source may restrict the generalizability of our findings to broader or community-based populations. In particular, the sample was composed exclusively of neurology inpatients, which may further limit the generalizability of our findings to broader, community-based populations. Furthermore, because all participants were Han Chinese inpatients from a single tertiary hospital, the study lacks ethnic and socioeconomic diversity, which may further constrain the generalizability of the results. Moreover, patients with a history of stroke were not excluded, as the study aimed to reflect real-world inpatient populations; however, this may introduce heterogeneity and confounding in the interpretation of WMH-related risk factors. Additionally, despite incorporating various clinical and biochemical variables, the model does not account for genetic predispositions, lifestyle factors, or advanced imaging biomarkers, which could further refine risk prediction. Furthermore, data on migraine history were not systematically collected, which may be relevant given evidence linking migraine-especially with aura-to increased WMH risk. In addition, WMH volume and spatial distribution were not analyzed, which should be explored in future studies. Future research should focus on four key areas. First, prospective multicenter validation is needed to confirm the model’s applicability across diverse populations. Second, incorporating additional risk factors, including genetic, lifestyle, and advanced imaging parameters (e.g., diffusion tensor imaging and perfusion MRI), may enhance predictive accuracy. Third, longitudinal studies could provide deeper insights into how these predictors influence WMH progression over time. Lastly, integrating this model into routine clinical practice and evaluating its impact on decision-making and patient outcomes remains a crucial area for further investigation.

In conclusion, while our study aligns with and extends existing literature, offering new insights into the relationship between WMHs and systemic health conditions, it also lays the groundwork for future research and clinical applications. Despite its limitations, this study contributes a valuable tool for risk assessment and early detection of WMH, paving the way for improved management of CSVD.

## Conclusion

In this study, we developed a predictive model for WMH by integrating diverse clinical and laboratory parameters. The model, incorporating key factors such as age, stroke history, hypertension, triiodothyronine levels, albumin-globulin ratio, and homocysteine levels, demonstrated robust performance in assessing WMH risk. Our findings provide novel insights into the interplay between systemic health and cerebral microvascular health, highlighting the importance of thyroid function and systemic inflammation in WMH pathophysiology.

Despite limitations such as its retrospective design and single-center data source, this study represents a valuable step toward improving early identification and management of WMH. Future research should focus on validating the model in diverse populations and exploring additional risk factors, such as genetic and lifestyle elements, to enhance its predictive accuracy.

In summary, this study offers a practical tool for WMH risk assessment, with the potential to improve prevention and management strategies in patients with cerebral small vessel disease.

## Data Availability

The original contributions presented in the study are included in the article/supplementary material, further inquiries can be directed to the corresponding author.
